# Increased levels of α2-3- and α2-6-linked sialic acids during airway inflammation govern influenza A binding to peripheral airway mucins in a subtype-dependent manner

**DOI:** 10.3389/fimmu.2026.1768280

**Published:** 2026-03-13

**Authors:** John Benktander, Macarena Paz Quintana Hayashi, Rickard Nordén, Lisa Pettersson, Magnus Paulsson, Anders Lindén, Sara K. Lindén

**Affiliations:** 1 Department of Medical Biochemistry and Cell Biology, Institute of Biomedicine, Sahlgrenska Academy, University of Gothenburg, Gothenburg, Sweden; 2 Department of Clinical Microbiology, Sahlgrenska University Hospital, Gothenburg, Sweden; 3 Department of Infectious Diseases, Institute of Biomedicine, Sahlgrenska Academy, University of Gothenburg, Gothenburg, Sweden; 4 Department of Clinical Microbiology, Umeå University, Umeå, Sweden; 5 Infection Medicine, Department of Clinical Sciences Lund, Faculty of Medicine, Lund University, Lund, Sweden; 6 Department of Clinical Microbiology, Skåne University Hospital, Lund, Sweden; 7 Division for Lung and Airway Research, Institute of Environmental Medicine, Karolinska Institutet, Stockholm, Sweden; 8 Karolinska Severe COPD Center, Department of Respiratory Medicine and Allergy, and Center for Molecular Medicine, Karolinska University Hospital, Stockholm, Sweden

**Keywords:** BAL, influenza A virus, inhibition, lung, mucins, O-glycan

## Abstract

**Background:**

Influenza infection increases the risk of pneumonia and respiratory failure in chronic obstructive pulmonary disease (COPD) and mucins play an important role in pulmonary host defense.

**Methods:**

Lower airway mucins were obtained from long-term smokers with and without COPD, pneumonia patients, and healthy never-smokers; oral MUC5B was obtained from a healthy never-smoker, and the ability of lower airway mucins and oral MUC5B to bind influenza A virus (H1N1 and H3N2) was investigated.

**Results:**

Lower airway mucins from long-term smokers with and without COPD, as well as the oral MUC5B, bound to H1N1. In contrast, all mucins, regardless of donors, bound to H3N2. Differences in binding were linked to more pronounced glycan sialylation in lower airway mucins from pneumonia patients and long-term smokers compared with healthy never-smokers. For lower airway mucins, the abundance of α2-6-linked NeuAc correlated with H1N1 binding, whereas the abundance of α2-3-linked NeuAc correlated with H3N2 binding. A neuraminidase inhibitor increased virus binding, even more so for H1N1 than for H3N2, resulting in the binding of both H1N1 and H3N2 to all mucins. The H1N1 neuraminidase cleaved α2-3- and α2-6-linked NeuAc to a similar degree, whereas the H3N2 neuraminidase mainly cleaved the α2-6-linked NeuAc. Mucins inhibited influenza A infection in a concentration-dependent manner in airway epithelial cells, although more so for H1N1 than for H3N2.

**Conclusion:**

Mucins inhibit influenza infection, and this effect depends on viral subtype. Neuraminidase inhibitors enable mucins with low sialic acid content to preserve their virus-binding ability, thereby underscoring their therapeutic potential.

## 1 Introduction

Chronic obstructive pulmonary disease (COPD) is the fourth (1) leading cause of death worldwide, with over 3 million deaths in 2019. Smoke from burned biomass, including tobacco, and particulate matter in the outdoor environment are known causes of COPD. Common symptoms include general fatigue, effort-related dyspnea, and coughing, often with phlegm (2). This phlegm is associated with poor prognosis, especially when it is excessive in concomitant chronic bronchitis. Tissue destruction and remodeling also occur in COPD, especially in concomitant emphysema. Current therapy provides relief from symptoms, but it has poor efficacy, in part because it has mainly been developed for asthma, with a different inflammatory endotype. Notably, patients with COPD have an estimated 1,770-fold higher risk of pneumonia and a 1,097-fold increased risk of respiratory failure after influenza infection, compared to patients with COPD without influenza infection, but the mechanisms are poorly understood (3).

Influenza virus is an RNA virus of the *Orthomyxoviridae* family, and there are four types of influenza viruses: A, B, C, and D. Of these, only A and B viruses are known to cause seasonal epidemics, with the former being the more common culprit. The influenza A virus is further divided into subtypes based on its cell surface proteins hemagglutinin (H) and neuraminidase (N) (4). Only a few of the subtypes largely affect humans, such as the current seasonal influenza A virus H1N1pdm09 and H3N2 that infects approximately a billion people and account for approximately half a million deaths on an annual basis (5).

For influenza viruses, the hemagglutinin is responsible for helping the virus attach to the host cell, thereby enabling the entry into the cell. Generally, influenza A of avian or equine origin binds sialic acid [i.e., *N*-acetyl neuraminic acid (NeuAc)] with an α2–3 linkage to galactose, while human influenza A preferably binds to sialic acids with an α2–6 linkage (6). However, this is a simplification, as the influenza A virus H1N1pdm09 that prefers binding NeuAcα2-6 may also have a significant binding to NeuAcα2-3 (7). In addition, the binding to the sialic acids may be affected by sialic acid type, the length of the glycan, the glycosidic bond of the Gal–GlcNAc linkage, and the sulfation/fucosylation of the glycan structures (8).

Neuraminidase is an exo-glycosidase that facilitates the cleavage of the α-ketosidic linkage of the sialic acids, which is important for the virus release from the cell membrane (9). However, it has been shown that neuraminidases such as N1, N2, and N9 also have sialic acid-binding functions (10); e.g., influenza A virus H11N9 [strain A/tern/Australia/G70C/75(H11N9)] agglutinates animal erythrocytes by the secondary receptor binding site referred to as a hemadsorption site (11). The neuraminidases, like the hemagglutinins, display differences in their preferred sialic acid linkage. For human influenza A viruses, the neuraminidase cleaves both α2-3- and α2-6-linked sialic acids (12, 13). The distribution of the host sialic acid receptors in humans shows mainly α2-6-linked glycans in the upper respiratory tract, while a more even distribution between α2-3- and α2-6-linked sialic acids is found in the lower respiratory tract, such as the trachea, bronchus, and lungs (9).

Mucus secreted into the airways is an important part of the anti-viral host defense, and the high sialic acid content of airway mucus has the potential to bind viruses to sialic acid-binding adhesins, limiting access to viral cell entry receptors (14). Neuraminidases may enable the influenza virus to pass through the mucus barrier by destroying these sialylated mucin receptor decoys; a balance between neuraminidase and hemagglutinin activity is needed for infection to occur (15). The main polymeric mucins making up the mucus in the airways are MUC5AC and MUC5B, and the membrane-bound mucin MUC1 is also present in the mucus due to shedding and the presence of alternatively spliced variants (16). When mucus becomes too thick, it can contribute to airway pathology. Previous studies on lower airway mucins (LAMs) show that long-term smokers (LTSs), both with and without COPD, have increased levels of MUC5AC and MUC1 compared to healthy never-smokers (HNSs) (17). Moreover, an increase in large MUC5AC complexes was also detected in LTSs, which was positively correlated with the level of blood leucocytes (17). MUC1 levels were positively correlated to bronchoalveolar lavage (BAL) leucocytes (17). Together, this suggests a link between mucus properties/composition and systemic and local inflammation. Notably, an increase in the MUC5AC to MUC5B ratio may be linked to the severity of COPD (18). Moreover, the relative abundance of α2-3- and α2-6-linked sialic acids is increased on MUC5 mucin O-glycans from LTSs with and without COPD compared to healthy non-smokers (19). Here, the increase in NeuAcα2–6 leads to an increased ability of the mucins to bind to the bacterial pathogen *Moraxella catarrhalis* (19). Additionally, the overexpression of MUC1 and the addition of short synthetic MUC1 *in vitro* decrease influenza A infection levels (20). The secreted MUC5AC mucin has also been shown to reduce the infection of influenza A virus H1N1 in Madin-Darby Canine Kidney (MDCK) cells and MUC5AC-transgenic mice with increased resistance to infection (21). Furthermore, MUC5B can cause the aggregation of influenza virus particles, especially in combination with the neuraminidase inhibitor oseltamivir (22).

For this study, we hypothesized that LAMs from patients with airway inflammation carry increased levels of α2-3- and α2-6-linked sialic acids, which in turn make these LAMs more efficient in binding to influenza A. Accordingly, the primary aim of this study was to characterize the binding of influenza A viruses (H1N1pdm09 and H3N2) to LAMs from LTSs with and without COPD, pneumonia patients, and HNSs. A secondary aim was to determine the impact of a neuraminidase inhibitor (oseltamivir) on neuraminidase activity and mucin binding. A tertiary aim was to address whether mucins are able to inhibit influenza infection *in vitro.*

## 2 Methods

### 2.1 Human samples

We obtained LAMs from BAL samples in two study materials, including the KOL-KB 2011 material and the Pneumonia 2016 material.

#### 2.1.1 KOL-KB 2011 material

The human donors for this material were recruited at Sahlgrenska University Hospital in Gothenburg after informed consent was obtained, in accordance with the recommendations of the *World Medical Association* (Ethical diary number 968-11; T136-13). This material included long-term smokers (LTSs, >20 pack-years) with (LTSs with COPD stage 1–4, n = 5) or without (LTSs, n = 9) COPD and healthy never-smokers (HNSs, n = 5) of both genders. The study participants were carefully characterized with respect to medical history, smoking, pulmonary comorbidities, ventilatory capacity before and after reversibility test, gas diffusion capacity, radiology, electrocardiogram, routine sputum bacteriology, and clinical chemistry tests, as previously described (17, 19, 23, 24). Pulmonary comorbidities other than chronic bronchitis and emphysema constituted exclusion criteria. All recruited subjects underwent bronchoscopy with bronchoalveolar lavage according to standard clinical protocols as previously described (17, 19, 23, 24). The key characteristics of this study material are summarized in **Supplementary Table 1** and **2**.

#### 2.1.2 Pneumonia 2016 material

The human donors for this material were recruited at Skåne University Hospital in Malmö after informed consent was obtained, in accordance with the recommendations of the *World Medical Association* (Ethical diary number 2016/523). Patients (LAM 1, n = 7, and LAM 2, n = 7) who displayed established clinical symptoms of pneumonia and radiological lung infiltrate(s) were characterized with respect to medical history, comorbidities, smoking, gas diffusion capacity, radiology, electrocardiogram, routine sputum bacteriology, and virology, as well as clinical chemistry tests as previously described (25, 26). They subsequently underwent bronchoscopy with BAL according to standard clinical protocols. The key characteristics of this study material are summarized in **Supplementary Table 3** and **4**.

MUC5B isolated from saliva from a healthy never-smoker was also included (oral MUC5B) with approval from the ethics review committee in Lund, Sweden (Dnr 2021-01781).

### 2.2 Isolation of mucins

The isolation of mucins was performed as previously described (17, 19, 27). Briefly, the BAL was pooled in their respective groups (HNSs, n = 5; LTSs, n = 9; LTSs with COPD, n = 4; pneumonia BAL1, n = 7; and pneumonia BAL2, n = 7) and subjected to five times its volume of extraction buffer (6 M GuHCl, 5 mM EDTA, 10 mM sodium phosphate buffer, pH 6.5, with 0.1 M PMSF) while homogenized in an Dounce homogenizer with four strokes with a loose pestle. The solution was then mixed slowly overnight (4 °C) and centrifuged (23,000 × *g* for 50 min at 4 °C). The soluble material was collected, and the insoluble pellets were re-extracted two times with 10 mL of the extraction buffer overnight while slowly mixing. The soluble fractions were pooled and then dialyzed twice against 10 volumes of extraction buffer. Extraction buffer was added to the samples until reaching 26 mL, and CsCl was added and dissolved in the solution while slowly stirring. The samples were transferred to Quick Seal ultracentrifuge tubes (Beckman Coulter, Brea, California) and filled up with 10 mM NaH_2_PO_4_, giving a starting density of 1.39 g/mL. After a density gradient centrifugation at 40,000 × *g* for 90 hours (15°C), fractions were collected from the bottom of the tube with a fraction collector. Fractions were analyzed for glycans and pooled accordingly. The fractions were coated onto a 96-well plate (Polysorb, NUNC A/S, Roskilde, Denmark) overnight (4°C), and a glycan assay based on aldehyde detection after periodate oxidation (previously described (28)) was used to identify the mucin-containing peak. The mucin-containing fractions were pooled, and mucin concentration was determined using the same assay.

### 2.3 Determination of mucin sialylation and sulfation levels

The glycosylation of the mucin samples was determined using liquid chromatography–mass spectrometry (LC-MS) as previously described (17, 19, 27). Raw MS data files can be found at https://glycopost.glycosmos.org/entry/GPST00014, http://doi.org/10.50821/GLYCOPOST-GPST000659, and https://glycopost.glycosmos.org/entry/GPST000163. Terminal epitopes/glycan structure was calculated as the sum of the relative abundance of glycan structures containing the epitopes (factoring multiple epitopes on the same glycan) divided by the total relative abundance of glycans.

### 2.4 Culture of influenza A

Two human Influenza A virus strains [A/Sweden/49/2020 (H1N1)pdm09 and A/Stockholm/30/2020 (H3N2)], hereafter referred to as H1N1 and H3N2, respectively, were propagated in MDCK-SIAT1 cells. These cells were stably transfected with the human CMP-N-acetylneuraminate β-galactoside α-2,6-sialyltransferase gene and overexpressed α-2,6-linked sialic acid receptors compared to parental MDCK cells (29).

MDCK-SIAT1 cells were maintained in growth medium consisting of Dulbecco's Modified Eagle Medium (DMEM) (Sigma, St. Louis, Missouri D6429) supplemented with 5% heat-inactivated fetal bovine serum (FBS), penicillin (100 U/mL)–streptomycin (100 µg/mL) (Sigma P0781), and Geneticin (100 µg/mL; Gibco, Grand Island, New York 11811-031). Geneticin was included in the medium to ensure the stability of the transfected plasmid and continued over-expression of the α-2,6-linked sialic acid receptor.

Virus growth medium (VGM) was used for infections and differed from growth medium in that FBS was omitted to avoid trypsin inactivation (trypsin is necessary for virus replication). VGM contained DMEM, penicillin–streptomycin, and 25 mM 4-(2-hydroxyethyl)-1-piperazineethanesulfonic acid (HEPES) (Invitrogen, Carlsbad, California 15630056).

The MDCK-SIAT1 cells were cultured to 80%–90% confluence. The cell layer was washed with 20 mL VGM one time prior to inoculation with the virus stock. The viral inoculum was absorbed at room temperature for 30 min, after which virus growth medium supplemented with TPCK-trypsin (stock 0.5 mg/mL; final concentration 2 µg/mL) was added (TPCK-treated trypsin enables the cleavage of the hemagglutinin protein, HA0, into HA1 and HA2, which is essential for the virus–host cell membrane fusion process during the initiation of infection). The virus-infected cell culture was incubated at 34°C. After 24–48 hours, when cytopathic effect (CPE) was clearly visible, the supernatant, including cell debris, was collected in Falcon tubes and gently centrifuged (200 × *g* for 10 min). The resulting supernatant was collected, aliquoted, and stored at −80°C. The pellets with cell debris were discarded. To determine the concentration, end-point titration was performed on MDCK-SIAT cells that were seeded in 96-well plates in VGM+TPCK-trypsin (2 μg/mL) medium and grown until 90% confluence. The viral stocks were diluted in growth medium in a 10-fold dilution series, and 60 μL from each dilution was applied to the cells. The cells were incubated at 34°C and 5% CO_2_ for 2 hours, after which an additional 60 μL VGM was added to each well. The infection was allowed to proceed for 48 hours at 34°C and 5% CO_2_. Thereafter, it was established at what given virus concentration an infection could be observed as plaques, using a light microscope. For both H1N1 and H3N2, the infectious virus concentration was 10–20 × 10^6^ infectious virus particles per mL.

### 2.5 Viral binding assay

Isolated mucins and references for α2-3- and α2-6-linked sialic acids (NeuAc) linked to human serum albumin [3′-sialyllactose-acetyl-phenylenediamine (ADP) human serum albumin (HSA) conjugate; degree of conjugation 22 mol/mol and 6′-sialyllactose-ADP HSA conjugate; degree of conjugation 15 mol/mol from IsoSep, Tullinge, Sweden] diluted to 6 µg/mL in 0.5 M GuHCl were coated onto a 96-well plate (Polysorb, NUNC A/S, Roskilde, Denmark) overnight (4°C). Wells were washed three times in Phosphate-Buffered Saline (PBS) with 0.05% Tween and then exposed to blocking reagent (1%) for ELISA (Roche, Basel, Switzerland) diluted in diH_2_O for 1 hour. Before adding the virus to the plate, the virus suspensions were preincubated for 30 min in blocking buffer with or without oseltamivir (30 nM) on ice. The blocking solution was removed, and 80 µL virus suspension in 1% blocking buffer with 0.05% Tween was added and incubated overnight (4 °C). Virus particles were diluted in blocking buffer and titrated to obtain a similar overall level of binding signal between the subtypes. This titration led to a final dilution of the H1N1 of 1:20 and H3N2 of 1:2, which, based on the end-point titration on MDCK-SIAT cells, corresponded to approximately 5 × 10^3^ H1N1 and 5 × 10^4^ H3N2 infectious virus particles/well. The wells were washed three times, then incubated with the primary antibody (anti-influenza A, AB10741, goat polyclonal, Sigma), and diluted 1:200 in blocking buffer for 1 hour. After washing the wells three times, the alkaline phosphatase conjugated secondary antibody [IgG [H+L)] anti-goat polyclonal; Fischer scientific, Waltham, Massachusetts], diluted 1:1,000 in blocking buffer, was added for 1 hour at room temperature. The wells were washed three times again, incubated in the dark for 3 hours with diethanolamine substrate (Medicago, Uppsala, Sweden AB), and read at 405 nm on a plate reader. For each mucin, a parallel assay containing all steps but with the virus omitted was included and used as the negative control for that sample.

### 2.6 In-Cell Western assay

The human adenocarcinoma cell line A549 (A549-CCL-185, isolated from the lung tissue of a white 58-year-old man with lung cancer, purchased from ATCC, Manassas, Virginia) was grown in F12/K medium (1150556; Gibco) with 10% FBS, 100 units/mL penicillin, and 100 µg/mL streptomycin in T75 cell culture flasks until near confluence. Cells were washed in PBS, and trypsin was added to the cells for approximately 5 min; 20 mL of cell culture media was added to the trypsinized cells and transferred to a 50-mL tube. The tube was centrifuged at 1,000 × *g* for 5 min. Then, the supernatant was discarded, and the cell pellet was resuspended in 10 mL of media. The cell concentration was calculated and diluted to 4 × 10^5^ cells/mL; 100 µL was added to each well of a 96-well cell culture plate (Nunc, optical bottom plate, black; Thermo Scientific, Waltham, Massachusetts). After 68 hours of culture at 37°C with 5% CO_2_, the infection was started: 20 µL of viral suspension (diluted 1:10 in F12/K medium, 100 units/mL penicillin, and 100 µg/mL streptomycin) was pretreated for 15 min with either a control or 0.3–10 mg/mL mucin. The control was composed of 0.412 M CsCl and 4 M guanidine chloride to match the concentration of these chemicals from the mucin density gradient fractions. Both control and mucin were sterile dialyzed four times against 2 M NaCl and then two times against PBS for at least 3 hours between each change before being added to the viral suspensions. The mucin and control solutions were added to 80 µL F12/K medium (without FBS, but with 100 units/mL penicillin and 100 µg/mL streptomycin). After replacing the cell culture media in the 96-well plate with the virus-containing medium, the plate was incubated at 37°C for 1 hour, and then the media were replaced with 100 µL F12/K medium with 2% FBS and antibiotics. After 24 hours of incubation at 37°C, the cell culture media were removed, and the cells were washed two times with PBS. Subsequently, 100 µL 4% paraformaldehyde (PFA) was added for 20 min at room temperature to fix the cells. The PFA was removed, and the wells were washed three times with PBS containing 0.05% Tween. The cells were permeabilized by adding 0.1% triton-X-100 in PBS (100 μL/well) for 20 min. After another three washes with PBS containing 0.05% Tween, the cells were incubated with the anti-influenza A primary antibody (MAB8251, mouse, polyclonal) diluted 1:1,000 in Intercept blocking buffer (Licor Biosciences, Bad Homburg, Germany). After 1 hour of incubation, cells were washed three times with PBS 0.05 Tween, and the secondary antibody (IRDye680RD, goat, anti-mouse IgG, 926-68070, LI-COR Biosciences) diluted 1:1,000 in Intercept blocking buffer (LI-COR Biosciences) was added. After 1 hour, the cells were washed three times in PBS containing 0.05% Tween, and then 1 µg/mL 4′,6-diamidino-2-phenylindole dihydrochloride (DAPI) in PBS was added to the wells for 5 min. Wells were finally washed with PBS three times, and then the DAPI signal at 460 nm and the Infrared (IR) signal at 700 nm were read on an Odyssey CLx.

### 2.7 Statistics

GraphPad Prism 10.2.0 was used to produce graphs and statistical analyses. BAL from each patient group was pooled (HNSs, n = 5; LTSs, n = 9; and LTSs with COPD, n = 4, pneumonia 1, n = 7, and pneumonia 2, n = 7) to obtain a sufficient amount of material for mucin isolation, glycomics, and downstream assays. The n thus refers to how many patient samples were pooled for each mucin isolation to obtain purified LAMs. The number of technical replicates used for the data produced in the figures is denoted “technical replicates” in the figure legends. The technical replicates were utilized to determine whether the binding signal of each sample was significantly different than the signal from the binding assay when performed in the absence of virus or in the presence of the neuraminidase inhibitor. Thus, the technical replicates were not used to compare the binding level between clinical study groups. Statistics used were ANOVA with Tukey’s multiple comparison, paired t-test, Pearson’s correlation, and unpaired t-test, as stated in the figure legends. p-Values ≤ 0.05 were considered significant.

## 3 Results

### 3.1 Sialylation and sulfation of mucin glycans

**Figure 1:**
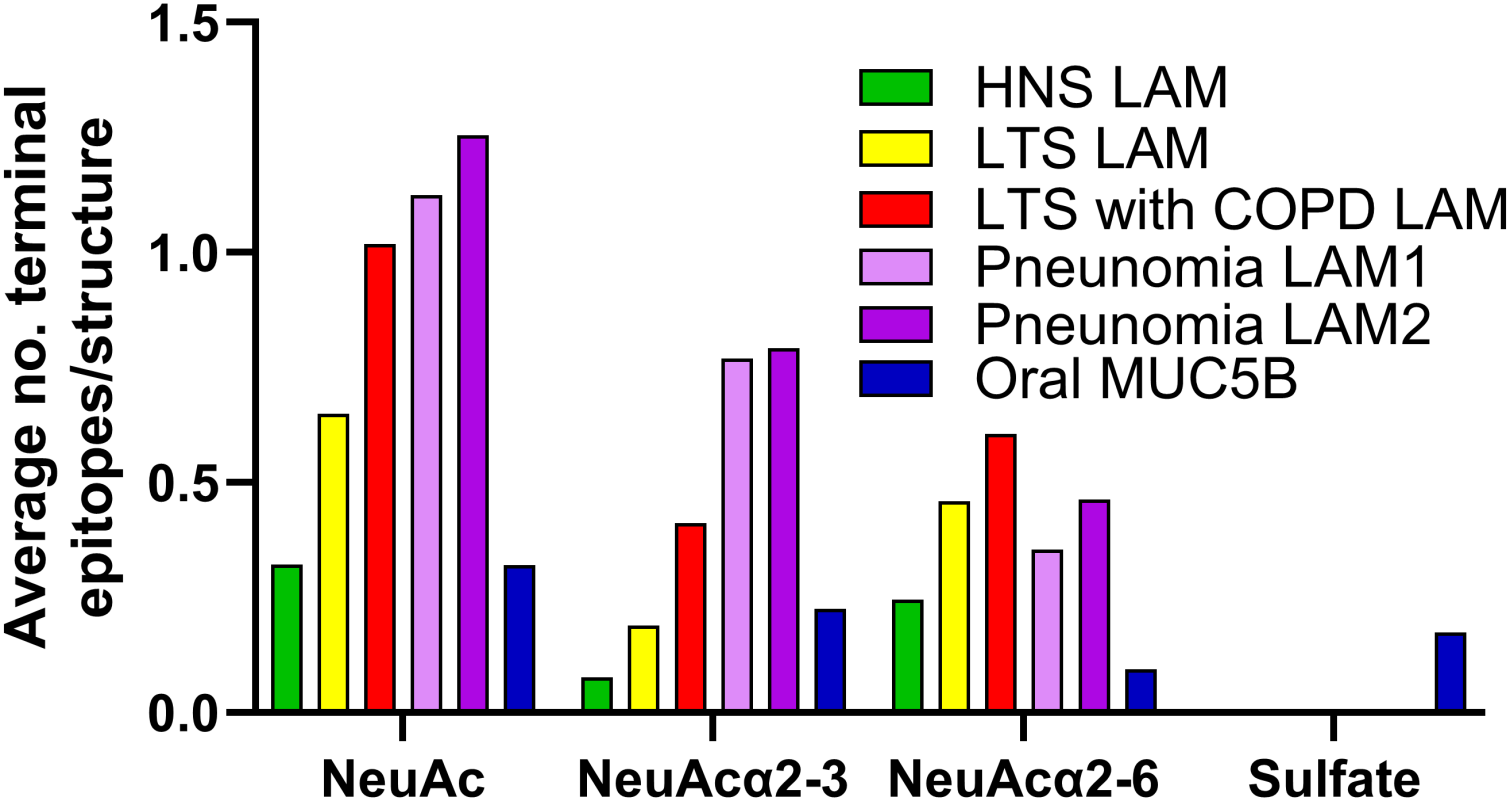
Acidic O-glycan moieties on lower airway and mucins (LAM) and oral MUC5B. Terminal epitopes/glycan structure were characterized by LC-MS and calculated as the sum of the relative abundance of glycan structure × number of epitopes on the structure in mucins pooled from healthy never-smokers (HNS LAMs, n = 5), long-term smokers (LTS LAMs, n = 9), LTSs with COPD (COPD LAMs, n = 4) and pneumonia patients (pneumonia LAM1, n = 7, and pneumonia LAM2, n = 7) as well oral MUC5B from a healthy individual. Since each glycan can have several epitopes (“branches”), the average can be above 1.

The LAMs were isolated from BAL samples pooled within the patient groups HNSs, LTSs without COPD, and LTSs with COPD, and from two pools from patients with pneumonia. The oral MUC5B was isolated from a salivary sample obtained from a never-smoker with no oral health problems. These materials have been described in previous studies in detail (17, 19, 27). Since sialic acid is important for interactions with influenza viruses, the relative abundance of acidic epitopes in these samples, based on LC-MS, was summarized (**Figure 1**). The sialic acid content in mucin pools from LTSs and all airway afflictions was higher compared to that of HNSs and oral MUC5B. Mucins pooled from HNS had the lowest (0.08) number of α2-3-linked sialic acid/glycan structures, while mucins pooled from patients with pneumonia contained the most (pneumonia pool 1 = 0.77, pneumonia pool 2 = 0.79; **Figure 1**). α2-6-Linked sialic acid was also relatively low in the HNSs (0.25) and oral MUC5B (0.09), with the highest number of α2-6-linked sialic acid/glycan found in the mucin pool from LTSs with COPD (0.61, **Figure 1**). The oral MUC5B contained similar levels of NeuAc as the airway mucins from HNS but higher levels of sulfated glycans (0.17), a feature that otherwise was found only in very low amounts in the pneumonia groups (<0.003, **Figure 1**).

### 3.2 Influenza A binding to mucins from patient groups and effect of a neuraminidase inhibitor

The ability of mucins to bind the influenza A virus types H1N1 and H3N2 was investigated using a microtiter plate-based assay. The assay in the presence of the neuraminidase inhibitor oseltamivir was performed to mimic the situation in airways treated with the anti-viral drug oseltamivir, whereas the assay in the absence of oseltamivir was used to mimic the situation in the untreated airway, where both the neuraminidase and the hemagglutinin act simultaneously.

**Figure 2:**
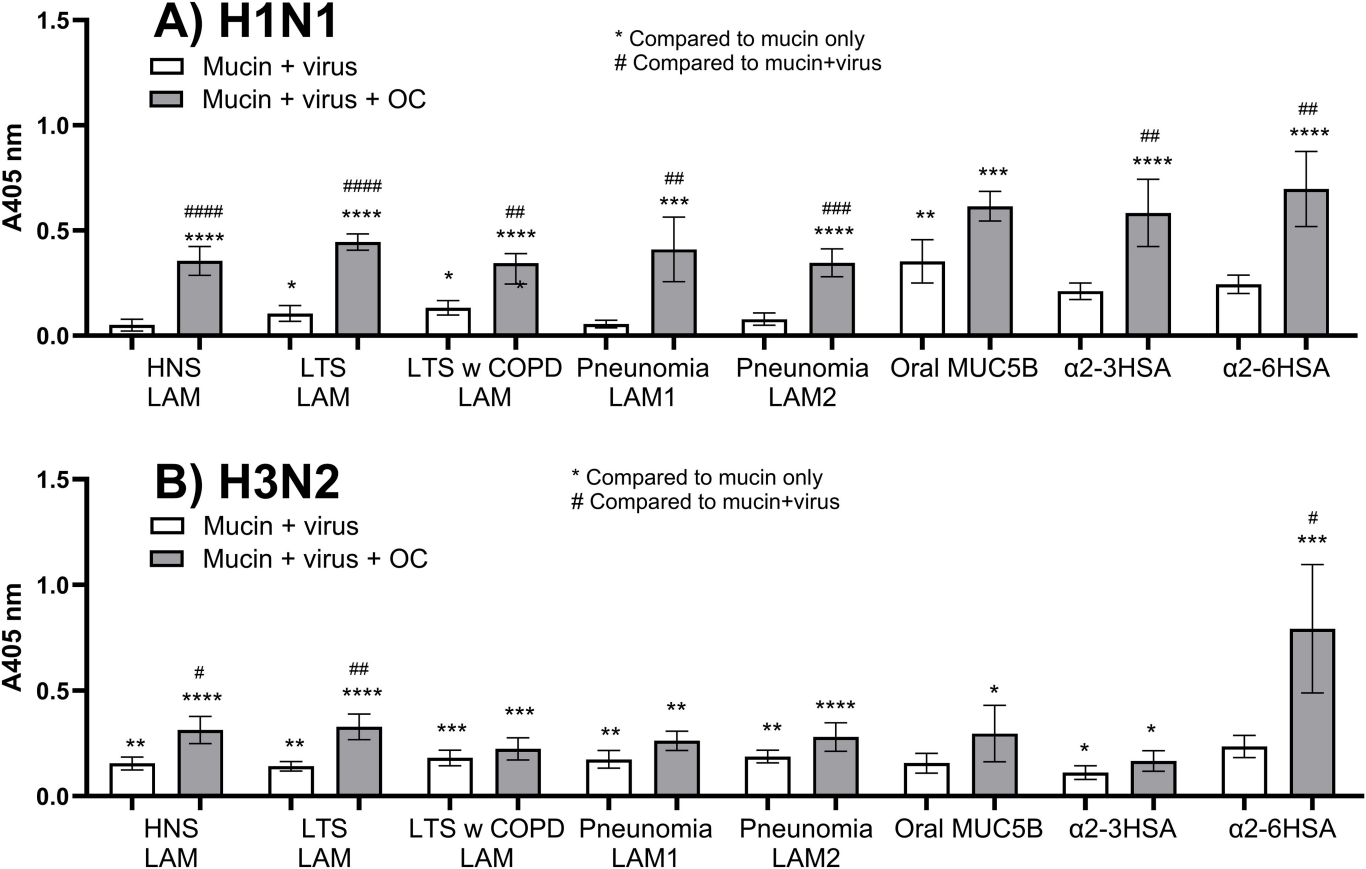
H1N1 and H3N2 binding to mucins and references in the absence and presence of oseltamivir. H1N1 **(A)** and H3N2 **(B)** binding was investigated using 6 µg lower airway mucins (LAMs) pooled from healthy never-smokers (HNS LAMs, n = 5), long-term smokers (LTS LAMs, n = 9), LTSs with COPD (COPD LAMs, n = 4), pneumonia patients (pneumonia LAM1, n = 7, and pneumonia LAM2, n = 7), and oral MUC5B from a healthy never-smoker (n = 1) as well as the references α2-3-HSA and α2-6-HSA linked NeuAc in a microtiter-based assay. Values are shown after subtracting the background signal from the same assay and mucin, but without added virus. Oseltamivir (30 nM) was added to the assay, forming the basis for the gray bars. Statistics were calculated using ANOVA with Tukey’s multiple comparison. Technical replicates, 5–12. Bars display means ± SEM. p: <0.05: *, <0.01: **, <0.001: *** and <0.0001: **** when compared to mucin only signal, while p: <0.05: #, <0.01: ##, <0.001: ### and <0.0001: #### when compared to mucin+virus signal.

In the presence of oseltamivir, both H1N1 and H3N2 bound to all mucins investigated as well as to the glycoconjugate references NeuAc linked α2–3 or α2–6 to human serum albumin (α2-3-HSA and α2-6-HSA, **Figure 2**). In the absence of oseltamivir, H1N1 binding to the α2-3-HSA or α2-6-HSA was not statistically significant (**Figure 2A**). Neither was H1N1 binding significant to mucins from the pool of HNSs or the two pools of mucins from patients with pneumonia; binding was detected to mucins from LTSs with and without COPD, as well as to oral MUC5B (**Figure 2A**). In contrast, H3N2 binding was detected to mucins from all sources and the α2-3-HSA (p < 0.05) but not to the α2-6-HSA reference (**Figure 2B**). Thus, in the absence of a neuraminidase inhibitor, influenza A binding to mucins differed between subtypes and mucins originating from patient groups, whereas in the presence of a neuraminidase inhibitor, the binding was similar.

### 3.3 Subtype-dependent effect of neuraminidase inhibition on mucin binding

**Figure 3:**
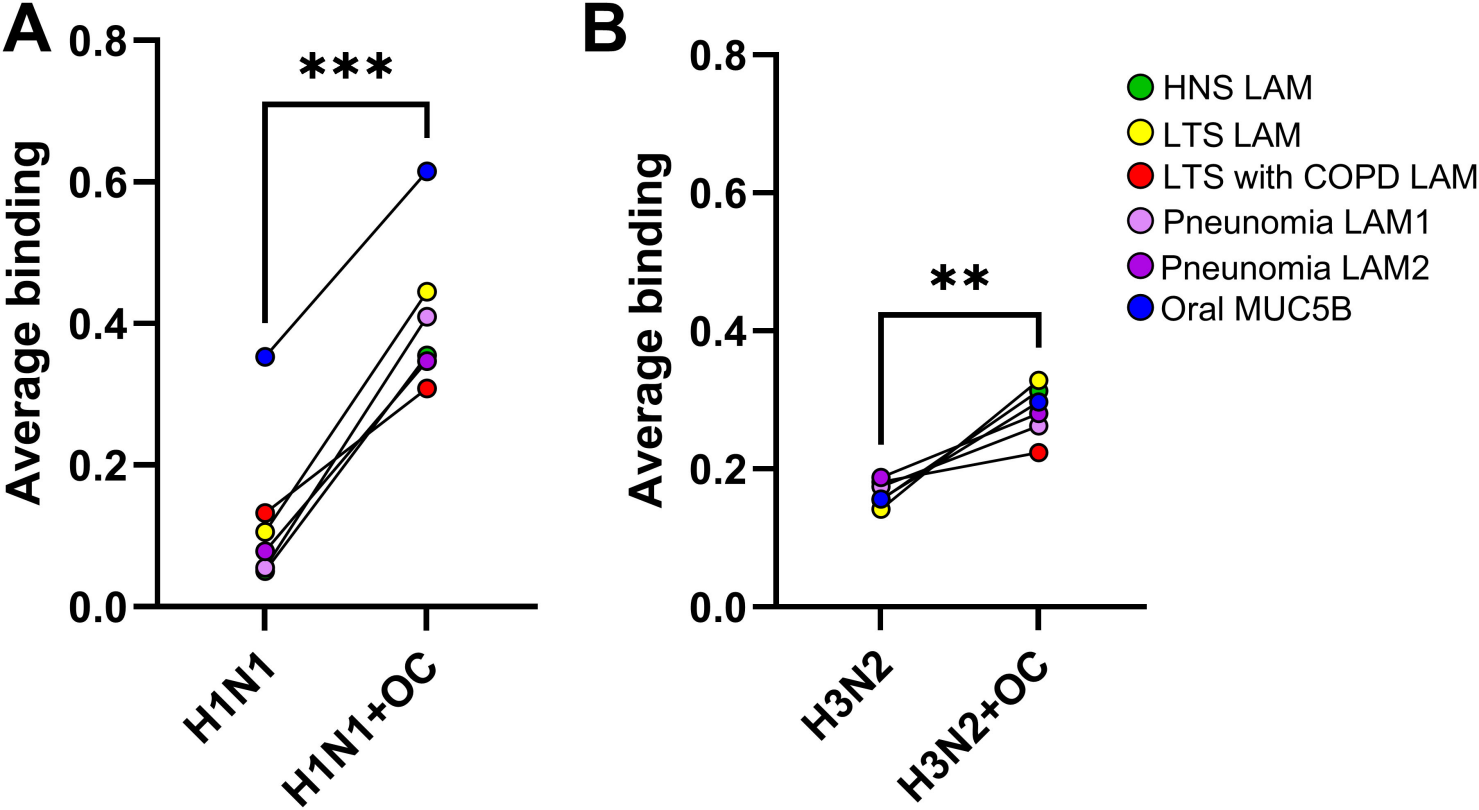
Effect of neuraminidase inhibition on the binding of influenza virus to mucins. Effect of oseltamivir (OC) on the binding of influenza A H1N1 **(A)** and H3N2 **(B)** to 6 µg lower airway mucins (LAM) pooled from healthy never-smokers (HNS LAMs, n = 5), long-term smokers (LTS LAMs, n = 9), LTSs with COPD (COPD LAMs, n = 4), pneumonia patients (pneumonia LAM1, n = 7, and pneumonia LAM2, n = 7), and oral MUC5B. Dots show the average binding signal from 11–12 technical replicates. Statistics: paired t-test. p: **<0.01, ***<0.001.

The level of H1N1 binding in the presence of oseltamivir was significantly higher compared to binding in the absence of oseltamivir to all mucins from the lower airways, and a similar trend was found for the oral MUC5B (**Figure 2A**). On average, the mucin binding level for H1N1 increased 4.5-fold (SEM: 0.96, p < 0.001) with the addition of oseltamivir (**Figure 3A**). H3N2 bound mucins to a more similar degree in the absence and presence of oseltamivir, with an average 1.7-fold (SEM: 0.16, p < 0.01) increase in the level of binding (**Figure 3B**). Thus, neuraminidase inhibition had stronger effects on the binding ability of H1N1 than H3N2.

### 3.4 Specificity of H1N1 and H3N2 neuraminidases

With the assumption that the reference HSA-linked sialic acids could be used as a measure for the activity of the neuraminidase, the effect of oseltamivir on mucin binding was calculated for both influenza A types. In the absence of oseltamivir, the H1N1 binding to α2-3-linked NeuAc was on average 62.8% lower (p < 0.05, unpaired t-test) compared to that in the presence of oseltamivir, while the α2-6-linked NeuAc was 64.9% lower (p < 0.01, **Figure 2A**). This demonstrated that the H1N1 neuraminidase was able to cleave sialic acids of both types at similar rates. For H3N2, no significant effect of oseltamivir was detected for α2-3-linked NeuAc, while binding to α2-6-linked NeuAc was 70.3% lower in the absence of oseltamivir (p < 0.05, **Figure 2B**), demonstrating that the H3N2 neuraminidase mainly cleaved the α2-6-linked sialic acid. Thus, the H1N1 neuraminidase cleaved α2-3- and α2-6-linked NeuAc at similar rates, whereas the H3N2 neuraminidase mainly cleaved the α2-6-linked NeuAc.

### 3.5 Correlation between influenza A binding and abundance of sialic acid epitopes

**Figure 4:**
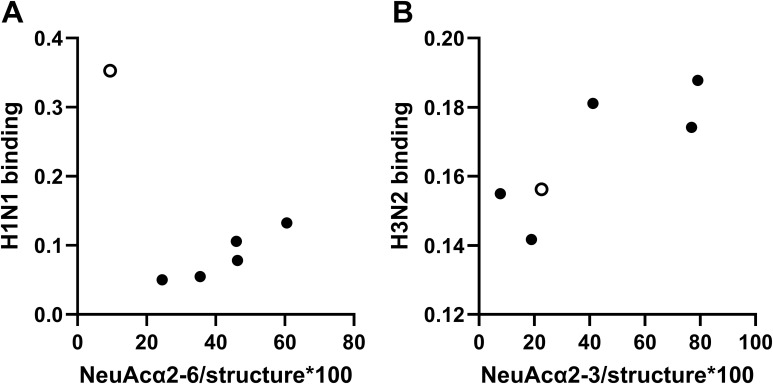
Correlation between binding of influenza A to mucins and abundance of sialylated mucin glycan epitopes. Correlation between influenza A binding to mucins from pooled lower airway mucins (LAMs) from healthy never-smokers (HNSs, n = 5), long-term smokers (LTSs, n = 9), LTSs with COPD (COPD, n = 4), pneumonia patients (pneumonia 1, n = 7, and pneumonia 2, n = 7), and oral MUC5B and the relative abundance of sialylated epitopes. Black dots show the average binding signal from 11–12 technical replicates of each LAM pool, whereas the white dot indicates oral MUC5B. **(A)** Correlation between the level of H1N1 binding and NeuAcα2-6/glycan structure on LAMs. Pearson’s correlation (binding to oral MUC5B, white dot, excluded): r = 0.930, p (two-tailed): 0.022. **(B)** Correlation between the level of H3N2 binding and NeuAcα2-3/glycan structure on LAMs and oral MUC5B. Pearson’s correlation: r = 0.813, p (two-tailed): 0.049.

Pearson’s correlation coefficients were calculated to investigate if there was a clear link between the relative abundance of total NeuAc, NeuAcα2-3, or NeuAcα2-6-linked to mucins and the level of influenza virus binding to the mucins. When including both LAMs and oral MUC5B, the H1N1 binding level did not statistically significantly correlate to NeuAc total, NeuAcα2-3, or NeuAcα2-6, as the level of binding to the oral MUC5B was notably higher than to the LAMs. However, when the oral MUC5B was excluded, a statistically significant correlation was present with the relative abundance of NeuAcα2–6 in the LAMs (**Table 1**; **Figure 4A**). The levels of H3N2 virus binding to mucins and α2-3-linked sialic acids and total sialic acid level on the mucins correlated (**Table 1** and **Figure 3B**). The level of H3N2 binding to the oral MUC5B mucin was similar to the level of the binding of LAMs with similar levels of α2-3-linked sialic acids (**Figure 4B**). Thus, α2-6-linked NeuAc on airway mucins correlated with H1N1 binding, whereas the abundance of α2-3-linked NeuAc and total NeuAc on all mucins correlated with H3N2 binding.

**Table 1:** Pearson’s correlation r values between virus binding and level of NeuAc type/structure.

Sialic acid	H1N1*	H3N2
Total NeuAc	0.215 (p: 0.729)	0.810 (p: 0.051)
NeuAcα2-3	−0.133 (p: 0.831)	**0.813 (p: 0.049)**
NeuAcα2-6	**0.930 (p: 0.022)**	0.456 (p: 0.362)

*The data on the oral MUC5B were not included. Bold numbers signify significant a correlation.

### 3.6 Inhibitory effect of mucins on influenza A infection in airway epithelial cells

The alveolar epithelial cell line A549 was infected with H1N1 and H3N2 influenza A virus, respectively, in the presence and absence of mucin and analyzed using In-Cell Western. Mucins isolated from the patient samples did not have sufficiently high concentration to use as an inhibitor in this experiment; a pooled fraction of pig colon mucins isolated previously (30) was used due to their high concentration and diverse glycosylation. A calculated 13% of the O-glycans were sialylated, with 1.7% NeuAc and 11.5% NeuGc. The relative abundances of α2-3- and α2-6-linked sialic acids were estimated to be 1.2% NeuAcα2-3, 0.5% NeuAcα2-6, 10.3% NeuGcα2-3, and 1.2% NeuGcα2-6. Furthermore, 34% of the structures were sulfated.

**Figure 5:**
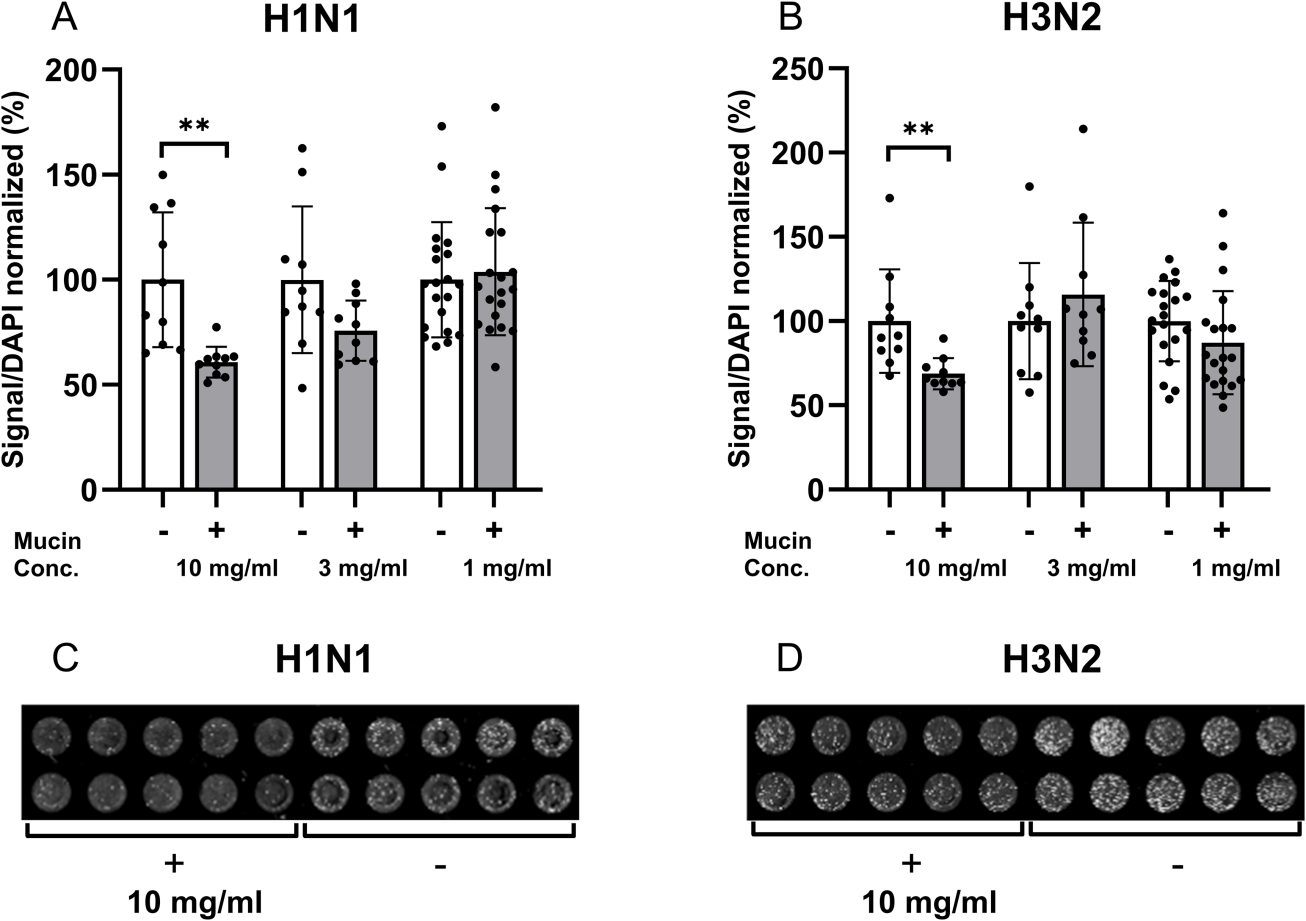
Inhibition of influenza A virus infection in an alveolar cell line by mucins. The 700-nm IR signal elicited by the antibodies detecting the virus, divided by the DAPI signal, was used as a measure of infection in A549 cells. The samples were normalized using the average infection signal of the cells infected in the absence of mucins as 100% to allow pooling of results from several experiments. **(A)** Quantification of H1N1 infection in the absence and presence of mucins. **(B)** Quantification of H3N2 infection in the absence and presence of mucins. **(C)** 700-nm IR detection of H1N1 infection in the absence and presence of mucins in an In-Cell Western assay. **(D)** 700-nm IR detection of H3N2 infection in the absence and presence of mucins in an In-Cell Western assay. Data are presented as mean (SEM), and p-values are based on unpaired t-tests. p: 0.01<**.

For the H1N1 influenza A virus, the infection level (the signal for antibody detection of the virus divided by the signal for the cell density) decreased on an average of 39.3% (SEM: 10.4, p < 0.01) in the presence of 10 mg/mL mucin compared to the control (**Figures 5A, C**). In the presence of 3 mg/mL mucin, the H1N1 signal trended toward a decrease of 24.3% (SEM: 11.9, p = 0.057), whereas 1 mg/mL mucin did not affect the level of infection (**Figure 5A**).

For the H3N2 influenza A virus, the infection level decreased by an average of 31.2% (SEM: 10.2, p < 0.01) in the presence of 10 mg/mL mucin compared to the control (**Figures 5B, D**). No significant effects were detected for 3 and 1 mg/mL mucin (**Figure 5B**). Thus, mucins inhibited *in vitro* influenza A infection in a subtype- and dose-dependent manner.

### 3.7 Impact of neuraminidase on the ability of mucins to inhibit influenza A infection in airway epithelial cells

**Figure 6:**
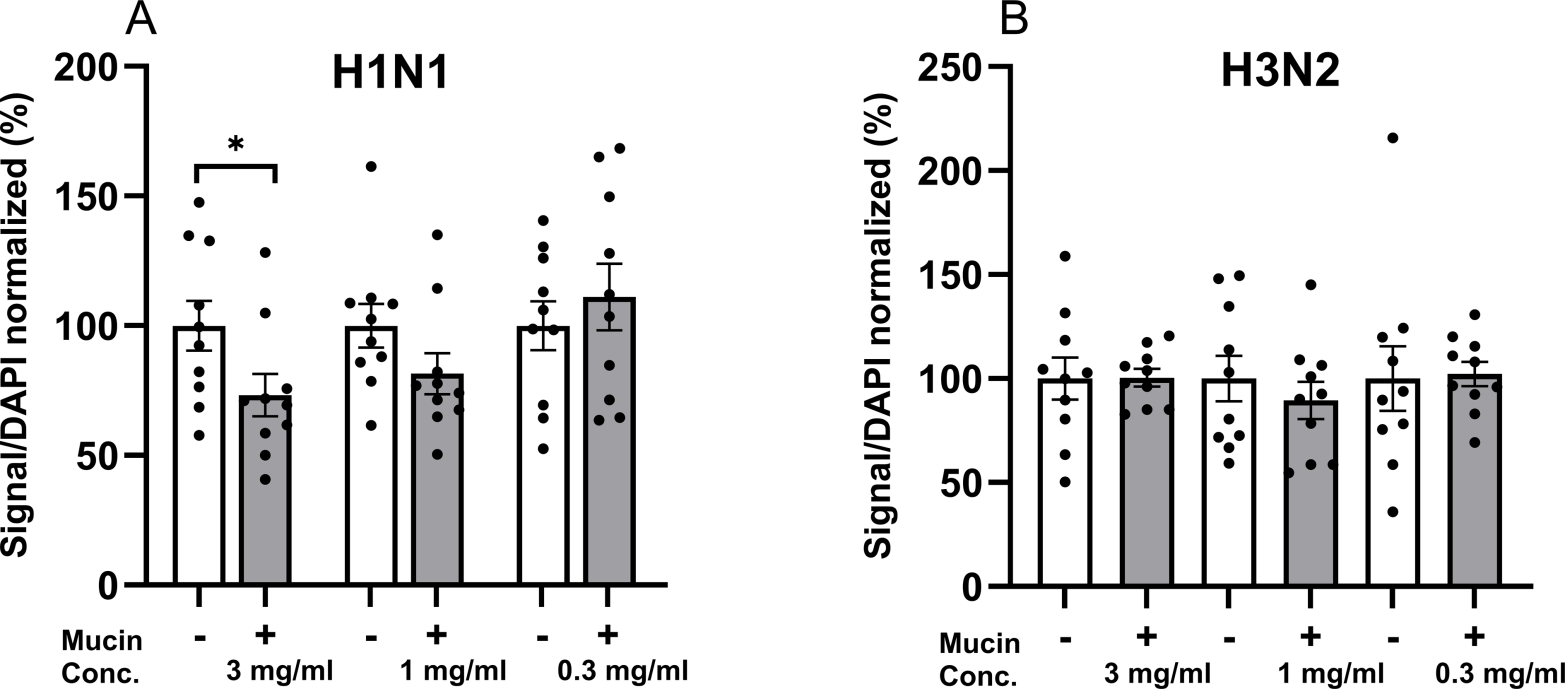
Inhibition of influenza A infection in an alveolar cell line by mucins in combination with oseltamivir. The 700 nm IR signal elicited by the antibodies detecting the virus, divided by the DAPI signal, was used as a measure of infection in A549 cells. The samples were normalized using the average infection signal of the cells infected in the absence of mucins as 100% to allow pooling of results from several experiments. **(A)** Quantification of H1N1 infection in the absence and presence of mucins **(B)**. Quantification of H3N2 infection in the absence and presence of mucins. Data are presented as mean (SEM), and p-values are based on unpaired t-tests.

Repeating the mucin inhibition assay in the presence of oseltamivir showed that H1N1 infection was inhibited with 3 mg/mL mucin by on average 26.8% (p < 0.05, **Figure 6A**), and a trend toward inhibition was present at 1 mg/mL too (p = 0.13, **Figure 5A**). H3N2 infection, however, showed no inhibition between the tested 0.3–3 mg/mL in combination with oseltamivir (**Figure 6B**). Thus, the level of inhibition from pig colon mucins was similar to the assay performed in the absence of oseltamivir.

## 4 Discussion

In this translational study on the mucin binding of influenza virus in airway inflammation, we found that the influenza A virus subtype H1N1 bound to LAMs from LTSs with and without COPD and oral MUC5B from a healthy non-smoker. In contrast, the subtype H3N2 bound to all investigated mucins. Interestingly, the level of H1N1 binding correlated with that of α2-6-linked sialic acid, while the level of H3N2 binding correlated with that of α2-3-linked sialic acids. The neuraminidase inhibitor oseltamivir caused an increased binding to mucin samples, which was more apparent for H1N1 than for H3N2. Furthermore, the H1N1-associated neuraminidase cleaved both α2-3- and α2-6-linked sialic acids, whereas the H3N2 neuraminidase mainly cleaved the α2-6-linked sialic acid. Mucins inhibited influenza A infection in A549 cells in a dose-dependent manner, and inhibition was more efficient for H1N1 than H3N2. The linkages and thereby three-dimensional presentation of the glycans differed between the mucin pools, allowing the investigation of the effects of the linkages on virus binding and neuraminidase activity in the context of the complex glycosylation present on human LAMs.

The removal of sialic acids by neuraminidase appears antagonistic to the hemagglutinin, as it may remove potential binding sites. However, the neuraminidase may also enable infection by cleaving the sialic acids on the protective mucin decoys, thus preventing the virus from being “rinsed” away with the mucus flow (31). Thus, for effective infection, it seems as if the hemagglutinin and the neuraminidase need to be in balance to enable binding to the cell membrane but not to the sialic acid-carrying mucin decoys (15). The addition of a neuraminidase inhibitor increased H1N1 binding to both references, indicating that the neuraminidase could cleave both α2-3- and α2-6-linked sialic acids. In contrast, the addition of a neuraminidase inhibitor only increased H3N2 binding to the α2-6-linked sialic acid reference, suggesting that the neuraminidase only cleaved NeuAcα2-6. The results from other studies show much variation in neuraminidase specificity due to differences in virus subtypes, substrate, and concentrations, and it has been suggested that the neuraminidase activity depends on host cell line and species (32). For example, egg and MDCK isolated influenza virus display a higher activity toward NeuAcα2-3, while the influenza virus isolated from Vero cells had a preference for NeuAcα2-6 (33). However, both the H1N1 and H3N2 used in the current study were produced in the same cell line (MDCK-SIAT1 cells), and therefore, the differences detected between H1N1 and H3N2 in the current study cannot be explained by differences in the origin of the host cell. Some studies have indicated that for human influenza A viruses, the neuraminidase cleaves both α2-3- and α2-6-linked sialic acids, while the neuraminidase originating from avian influenza viruses cleaves only α2-3-linked sialic acids (12, 13).

Previous studies have shown that human influenza A preferably binds to sialic acids with an α2–6 linkage (6), although H1N1pdm09, which prefers binding NeuAcα2-6, may also bind to NeuAcα2-3 (7). Here, we found that both H1N1 and H3N2 bound to NeuAc linked via both linkages, with trends toward higher binding to NeuAcα2–6 than NeuAcα2–3 in the presence of a neuraminidase inhibitor. Furthermore, mucins from all sources bound to both H1N1 and H3N2 in the presence of a neuraminidase inhibitor. However, in the absence of a neuraminidase inhibitor, the actions of the neuraminidase and agglutinin compete for the sialic acids. When we addressed the nature of the binding of mucins to the virus in the absence of neuraminidase, we found that the NeuAcα2–3 content on O-linked glycans on all mucins investigated correlated with binding to H3N2. This is in line with the above mentioned results that the H3N2 cleaved α2-6 linked sialic acid: removal of NeuAcα2–6 epitopes by the H3N2 neuraminidase leaves NeuAcα2–3 epitopes for the binding of the H3N2 agglutinate. In contrast, the H1N1 neuraminidase cleaved both α2-3- and α2-6-linked sialic acids, and the relative abundance of α2-6-linked NeuAc on LAMs correlated with binding to H1N1. This is in line with the fact that the NeuAcα2-6-HSA standard tended to bind more H1N1 than the NeuAcα2-3-HSA standard, but may also be linked to the presentation of the sialic acids, as the length of the glycan, the glycosidic bond of the Gal–GlcNAc linkage, and the sulfation/fucosylation of the glycan structures also can influence the binding (8). Notably, the binding of the oral MUC5B to H1N1 was much stronger than expected based on the oral MUC5B sialic acid levels. That is, the level of H1N1 binding to the LAMs correlated strongly with the level of α2-3-linked NeuAc, whereas the level of binding to the oral MUC5B was several-fold higher than what could be expected from the level of α2-3-linked NeuAc in the oral MUC5B sample. In contrast, the level of H3N2 binding correlated with the level of α2-6-linked NeuAc of all mucin samples in the study, including the oral MUC5B sample. A possible cause of the high H1N1 binding ability of oral MUC5B was that it had more than a 50-fold higher level of sulfation than the LAMs. It has previously been shown that some H1N1 strains exhibit a high affinity to sulfated glycans containing NeuAcα2-3Galβ-R (34). The relative abundance of sulfated O-glycans was 16.7% of the oral MUC5B used in the current study, whereof only 0.3% contained NeuAc, while 16.4% were fucosylated structures. Thus, it appears likely that sulfated glycans without sialylation can confer binding to H1N1.

Results showed that 10 mg/mL of pig colon mucins was enough to significantly inhibit both H1N1 and H3N2 infections of the alveolar epithelial cell line A549. Pig mucin at a concentration of 3 mg/mL also had a trend of inhibiting H1N1 infection (24.3%, p = 0.057) but not the H3N2. This difference may be due to the fact that these mucins, in addition to carrying sialic acid, also contained sulfated glycans, and sulfation increased the ability of the H1N1 to bind to the virus. We expected that the addition of oseltamivir would increase the ability of the mucins to inhibit infection; however, no clear effects on infection level from adding oseltamivir were detected. In the presence of oseltamivir, 3 mg/mL mucins inhibited H1N1 infection by 26.8% (p < 0.05). It has been shown that neuraminidase inhibition affects infection depending on the receptor-binding specificity of the hemagglutinin and the receptor repertoire on the cells (35). We interpret the lack of the effect of oseltamivir found in the current study to likely depend on the fact that the level of sialylation was relatively low, whereas the level of sulfation was relatively high on the pig colonic mucins. Thus, the inhibitory effect can vary depending on cell line, virus subtype, and inhibitory mucin used.

A limitation of the study is that it was not possible to set the virus particle number exactly (i.e., PCR quantifies inactive virus as well, whereas the infectious particle count is toward a particular cell line, and may therefore not be fully representative of the human airway); thus, exact comparisons between binding signals cannot be made between H1N1 and H3N2. It is thus possible that the stronger effect of oseltamivir on H1N1 binding was affected by differences in neuraminidase levels, however, the effect on the standards was in a similar range (65 vs. 70%, respectively), suggesting that the differences in the effect of neuraminidase inhibitor instead were due to the fact that the H1N1 neuraminidase had a broader specificity than the H3N2 neuraminidase. Another limitation was that the infection inhibition experiments were conducted using pig colon mucins instead of human LAMs. This substitution was necessary due to the difficulty of obtaining sufficiently large volumes of clinical samples. Even if adequate volumes had been available, the mucins would have required concentration to reach experimental thresholds. However, mucins are notoriously adhesive, and substantial losses often occur during concentration steps. Such losses can disproportionately affect specific mucin subsets, introducing variability and reducing patient-specific relevance of the assay. The pig mucins used in the infection-inhibition assays displayed markedly lower sialylation than both the human LAM pools and oral MUC5B, particularly compared to the elevated sialylation observed in LAM pools from patients with pneumonia and COPD. In contrast, the level of sulfation in the pig mucins was approximately twice that of oral MUC5B, and the difference in sulfation levels with the LAM pools was even more pronounced. Consequently, it appears likely that the inhibitory effect of mucins on H3N2 infection would have been higher if we had used mucins with a level of sialylation similar to that of the LAM pools. Furthermore, the effects observed may underestimate the true impact of oseltamivir for both H3N2 and H1N1 since its efficacy would likely be greater under the higher sialic acid densities characteristic of human LAMs.

Mucins normally have an important role in trapping pathogens, acting as decoys to prevent adhesion to the epithelial cells. Bound pathogens can then be removed by mucus shedding (36–38). As indicated by a multi-center study on induced sputum, smokers with COPD display a MUC5AC concentration that is 10 times higher and a MUC5B concentration that is three times higher compared to that of never-smoking controls (39). It can be argued that the results of the referred study were confounded by the inclusion of patients with the comorbidity asthma in up to 18% of the cases, but our own study on BAL samples from COPD patients without asthma also demonstrates that the concentration of MUC5AC and MUC1 is higher in LTSs with and without COPD than in never-smokers (17). Thus, together with the proportion of sialylated glycans being higher in the LTSs with and without COPD, the total concentration of sialylated mucin with potential to act as decoys for the cell-bound sialylated virus target is likely to be approximately 20-fold higher in the airways of LTSs compared to HNSs. Despite this theoretically beneficial response of pulmonary host defense, current smokers are more likely to develop laboratory-confirmed influenza than non-smokers (40). The findings related above may lead to the expectation that the increased levels of receptor decoys would decrease the risk of becoming infected, but LTSs with and without COPD also have a larger proportion of their MUC5AC in the form of large complexes (17). Moreover, the clearance of mucins is decreased in patients with COPD, leading to the potentially harmful accumulation of mucus in the airway (41). Thus, it seems likely that the effect of the pulmonary host defense in terms of increasing sialic acids in local mucins, which in healthy airways contribute to the clearance of the virus, is too limited to be sufficient in COPD due to hampered efficiency in the clearance of trapped pathogens. Clearly, the underlying molecular mechanisms constitute potential therapeutic targets and therefore deserve further exploration, even more so given that pathogenic alterations in airway mucins are relevant in several clinically important airway disorders beyond COPD and pneumonia (42).

In conclusion, as indicated in smoking, COPD, and pneumonia, local mucins display an increased level of sialic acids in airway inflammation. These increased levels of sialic acid on mucins, as well as the increased concentration of mucins found in the affected patients, increase the total binding potential of the mucin to influenza A virus for both H1N1 and H3N2. Due to this, we propose that the underlying molecular mechanisms offer therapeutic potential for reinforcing pulmonary host defense by enhancing sialylation in local mucins and thus their ability to bind to pathogens, in combination with treatments that decrease properties that are likely to contribute to airway obstruction, such as the increased proportion of large complexes found in LTSs (17).

## Data Availability

The datasets presented in this study can be found in online repositories. The names of the repository/repositories and accession number(s) can be found in the article/**Supplementary Material**.
