# Supplementary material for: Increased levels of α2-3- and α2-6-linked sialic acids during airway inflammation govern influenza A binding to peripheral airway mucins in a subtype-dependent manner
Source: Front Immunol. 2026 Mar 13;17:1768280. doi: 10.3389/fimmu.2026.1768280 (PMC13021647; doi:10.3389/fimmu.2026.1768280)
Supplement: Supplementary file 1 [file Table1.docx]

**Online data supplement**

**Increased levels of α2-3 and α2-6-linked sialic acid during airway inflammation govern influenza A binding to peripheral airway mucins in a subtype dependent manner**

John Benktander, Macarena Paz Quintana Hayashi, Rickard Nordén, Lisa Pettersson, Magnus Paulsson, Anders Lindén and Sara K. Lindén

**Supplementary Table 1***.* Demographics and clinical parameters of study subjects in KOL-KB 2011: never-smokers (n=5), smokers with (n=4) and without COPD (n=9). [1, 2].

| **Parameters** | **Study groups** | | | | |
| --- | --- | --- | --- | --- | --- |
|  | **Never-smoker** | | **Smoker** | | **COPD** |
| Number of subjects | 5 | | 9 | | 4 |
| Gender (M/F) | 4/1 | | 4/5 | | 2/2 |
| Age (years) | 45 (41-66) | | 67 (45-72) | | 57 (52-64) |
| Current smoking (cigarettes/day) | 0 (0-0) | | 10 (10-40) | | 14 (8-20) |
| Pack-years (years) | 0 (0-0) | | 38 (21-51) | | 32 (20-40) |
| FVC | 5.7 (3.5-7-0) | | 3.2 (2.5-5.4) | | 4.4 (3.5-6.3) |
| FVC (% predicted) | 123 (109-127) | | 116 (74-125) | | 119 (97-130) |
| FEV1 | 4.7 (2.6-5.1) | | 2.4 (2.0-3.9) | | 2.9 (2.1-3.2) |
| FEV1 (% predicted) | 108 (100-127) | | 100 (68-116) | | 81 (75-105) |
| FEV1/FVC | 74 (68-83) | | 73 (70-82) | | 61 (50-68) |
| LAS volume (mL) | 150 | | 150 | | 150 |
| LAS recovery (%) | 57 (25-74) | | 53 (32-68) | | 37 (22-60) |
| LAS total cell (X10^6^) | 10 (4-13) | | 24 (9-41) | | 16 (10-26) |
| Blood CRP (µg/mL) | 0.6 (0.44-0.72) | 1.5 (0.34-13) | | 2.2 (0.2-15) | |
| LAS Eosinophil (%) | 0 (0-0) | 0 (0-0) | | 2 (0-5) | |
| LAS Neutrophil elastase (ng/mL) | 178 (140-982) | 143 (80-254) | | 136 (117-5527) | |
| Blood leukocytes (10^9^ cell/L) | 5.2 (4.2-7) | 6.2 (5.2-9.7) | | 7.6 (6-8.1) | |
| Blood lymphocytes (10^9^ cell/L) | 1.8 (1.3-2.8) | 1.9 (1.5-2.9) | | 2.8 (1.8-3.2) | |
| Blood neutrophil (10^9^ cell/L) | 3 (2.3-3.6) | 3.6 (3.2-6) | | 3.8 (2.7-4.6) | |

**Supplementary Table 2***.* Bacteria grown from Bronchoalveolar lavage from study subjects in KOL-KB 2011: never-smokers (n=5), smokers with (n=4) and without COPD (n=9). These data have been included in previous publications [1, 2].

| **Patient group** | **Bacterial species** | **Quantity (CFU/mL)** |
| --- | --- | --- |
| Never-smoker | No bacteria | - |
| Never-smoker | No bacteria | - |
| Never-smoker | No bacteria | - |
| Never-smoker | Normal oral microflora | 4000 |
| Never-smoker | *S. pneumoniae/H. influenzae/α-Streptococcus* | 10,000/10,000/10,000 |
| Smoker | Normal oral microflora | 2000 |
| Smoker | Normal oral microflora | 121,000 |
| Smoker | Normal oral microflora | 12,000 |
| Smoker | Normal oral microflora | <100 |
| Smoker | Normal oral microflora | 300 |
| Smoker | Normal oral microflora | 3,000 |
| Smoker | No bacteria | - |
| Smoker | Normal oral microflora /*S. pneumoniae* | 2000/1000 |
| Smoker | No bacteria | - |
| COPD | No bacteria | - |
| COPD | No bacteria | - |
| COPD | No bacteria | - |
| COPD | Normal oral microflora | 100,000 |

**Supplementary Table 3***.* Demographics and clinical parameters of study subjects in Pneumonia 2017: Pneumonia LAM1 was isolated from BAL pooled from pneumonia 1 (n=7) and Pneumonia LAM2 was isolated from BAL pooled from pneumonia 2 (n=7).

| **Parameters** | Pneumonia 1 | Pneumonia 2 |
| --- | --- | --- |
| Number of subjects | 7 | 7 |
| Gender (M/F) | 3/4 | 3/4 |
| Age (years, average, range) | 65, 39-81 | 72, 39-83 |
| Smoking (currently/previously/never) | 0/1/4** | 0/2/5 |
| **Underlying conditions** |  |  |
| COPD (Yes/No) | 0/7 | 1/7 |
| Other pulmonary diseases (Yes/No) | 1/7 | 1/7 |
| **Current disease** |  |  |
| Community acquired pneumonia (Yes/No) | 2/5 | 1/6 |
| Nosocomical pneumonia (Yes/No) | 5/2 | 6/1 |
| Radiographic lung infiltrate (Yes/No) | 5/2 | 6/1 |
| Purulent secretions (Yes/No) | 3/2** | 5/2 |
| Temp >38°C within the last 24h (Yes/No) | 6/1 | 6/1 |
| Duration of symptoms (days, range) | 16, 2-34 | 11, 1-30 |
| Mechanical ventilator (Yes/No) | 3/4 | 4/3 |
| Days with ventilator | 2, 5, 7 | 5, 5, 3, 12 |
| **Laboratory data** |  |  |
| Plasma-CRP (µg/mL, average, range) | 96, 9-180 | 69, 15-168 |
| Blood leukocytes (10^9^ cell/L) | 12, 5-18 | 11, 4-16 |
| **Current medication** |  |  |
| Antibiotic treatment (Yes/No) | 7/0 | 7/0 |
| **missing data from 2 patients |  |  |

**Supplementary Table 4***.* Microbes detected in Bronchoalveolar lavage from study subjects in Pneumonia 2017: Pneumonia LAM1 was isolated from BAL pooled from pneumonia 1 (n=7) and Pneumonia LAM2 was isolated from BAL pooled from pneumonia 2 (n=7).

| **Pneumonia pool 1** |  | **Pneumonia pool 2** |  |
| --- | --- | --- | --- |
| Patient 1 | *Enterobacter cloacae, Aspergillus fumigatus* | Patient 8 | *Serratia marcescens* |
| Patient 2 | *Nocardia farcinica, Enterococcus faecium* | Patient 9 | *Pseudomonas aeruginosa* |
| Patient 3 | *Pseudomonas aeruginosa, Klebsiella pneumoniae* | Patient 10 | *Stenotrophomonas maltophila, Klebsiella pneumoniae* |
| Patient 4 | *Staphylococcus aureus* | Patient 11 | *Metapneumovirus* |
| Patient 5 | *Pneumocystis jiroveci* | Patient 12 | *Aspergillus fumigatus, Mycobacterium avium* |
| Patient 6 | *Legionella pneumophila,* Influenza A H1N1 | Patient 13 | *Pseudomonas aeruginosa* |
| Patient 7 | *Staphylococcus aureus* | Patient 14 | *Candida albicans, Enterococcus faecium* |

| **Supplementary Table 5. Average number of terminal epitopes/glycan. Data used for Figure 1.** | | | | | |  |
| --- | --- | --- | --- | --- | --- | --- |
|  | **HNS LAM** | **LTS LAM** | **LTS with COPD LAM** | **Pneunomia**  **LAM 1** | **Pneunomia**  **LAM 2** | **Oral MUC5B** |
| **NeuAc** | 0.322437 | 0.649083 | 1.017668 | 1.123828 | 1.254036 | 0.320116 |
| **NeuAcα2-3** | 0.077276 | 0.189607 | 0.411868 | 0.769114 | 0.791267 | 0.226017 |
| **NeuAcα2-6** | 0.245162 | 0.459477 | 0.6058 | 0.354714 | 0.462769 | 0.094099 |
| **Sulfate** | 0 | 0 | 0 | 0.002969 | 0.001826 | 0.174928 |

**Supplementary Table 6. H1N1 binding to 6 µg mucins. Data used for Figure 2A. Values in the Figure are shown after subtracting signal from mucins without added virus.**

| **HNS LAM** | -0.01 | -0.05 | 0.04 | 0.02 | 0.04 | -0.02 | -0.02 | 0.01 | 0.01 | -0.02 |  |  |
| --- | --- | --- | --- | --- | --- | --- | --- | --- | --- | --- | --- | --- |
| **HNS LAM+H1N1** | -0.02 | -0.12 | -0.03 | -0.01 | 0.07 | 0.08 | 0.11 | 0.24 | 0.11 | 0.06 | 0.06 |  |
| **HNS LAM+H1N1+OC** | 0.31 | 0.54 | 0.55 | 0.18 | 0.17 | 0.38 |  |  |  |  |  |  |
| **LTS LAM** | 0.03 | -0.08 | 0.03 | 0.02 | 0.04 | -0.03 | -0.01 | -0.01 | -0.02 | 0.03 |  |  |
| **LTS LAM+H1N1** | 0.33 | 0.02 | 0.11 | 0.07 | -0.06 | 0.03 | 0.03 | 0.08 | 0.06 | 0.14 | 0.07 | 0.39 |
| **LTS LAM+H1N1+OC** | 0.41 | 0.46 | 0.46 | 0.28 | 0.51 | 0.55 |  |  |  |  |  |  |
| **LTS COPD LAM** | 0.02 | -0.07 | 0.03 | 0.01 | 0.16 | -0.07 | -0.09 | 0.05 | 0.02 | -0.07 |  |  |
| **LTS COPD LAM+H1N1** | 0.11 | 0.09 | 0.1 | 0.11 | -0.05 | -0.04 | 0.17 | 0.11 | 0.36 | 0.11 | 0.21 | 0.31 |
| **LTS COPD LAM+H1N1+OC** | 0.38 | 0.31 | 0.32 | 0.05 | 0.42 | 0.37 |  |  |  |  |  |  |
| **Pneunomia LAM1** | 0.01 | -0.01 | 0 | 0.06 | -0.01 | -0.08 | 0.03 | 0.15 | -0.14 | -0.01 |  |  |
| **Pneunomia LAM1+H1N1** | 0.14 | 0.14 | 0.09 | 0.1 | 0.01 | 0.11 | 0.03 | 0.04 | 0.07 | -0.05 | 0.02 | -0.04 |
| **Pneunomia LAM1+H1N1+OC** | 1.08 | 0.42 | 0.51 | 0.04 | 0.09 | 0.32 |  |  |  |  |  |  |
| **Pneunomia LAM2** | -0.04 | 0.05 | -0.12 | 0.12 | -0.05 | 0.04 | 0 | 0 | -0.05 | 0.05 |  |  |
| **Pneunomia LAM2+H1N1** | 0.09 | 0.01 | -0.01 | 0.09 | 0.15 | 0.04 | 0.28 | 0.18 | -0.06 | 0 | -0.01 | 0.18 |
| **Pneunomia LAM2+H1N1+OC** | 0.48 | 0.39 | 0.54 | 0.09 | 0.32 | 0.26 |  |  |  |  |  |  |
| **Oral MUC5B** | 0.05 | 0.04 | -0.04 | -0.05 | -0.05 | 0.04 | 0 | 0.08 | -0.03 | -0.05 |  |  |
| **Oral MUC5B+H1N1** | 0.04 | 0.06 | 0.19 | 0.15 | 0.04 | 0.28 | 0.18 | 0.76 | 0.65 | 0.46 | 1.07 |  |
| **Oral MUC5B+H1N1+OC** | 0.73 | 0.44 | 0.38 | 0.72 | 0.61 | 0.81 |  |  |  |  |  |  |
| **α2,3-HSA** | -0.02 | 0.03 | -0.01 | 0.04 | -0.08 | -0.08 | -0.22 | 0.14 | -0.1 | 0.19 |  |  |
| **α2,3-HSA+H1N1** | 0.1 | 0.12 | 0.07 | 0.18 | 0.32 | 0.06 | 0.19 | 0.33 | 0.37 | 0.37 |  |  |
| **α2,3-HSA+H1N1+OC** | 0.33 | 0.5 | 0.35 | 0.23 | 0.84 | 1.25 |  |  |  |  |  |  |
| **α2,6-HSA** | -0.01 | 0.01 | 0 | -0.09 | 0.11 | -0.02 | -0.12 | 0.12 | 0 |  |  |  |
| **α2,6-HSA+H1N1** | 0.1 | 0.18 | 0.06 | 0.28 | 0.17 | 0.5 | 0.43 | 0.21 | 0.08 | 0.16 | 0.47 | 0.29 |
| **α2,6-HSA+H1N1+OC** | 0.55 | 0.53 | 0.83 | 1.52 | 0.36 | 0.39 |  |  |  |  |  |  |

**Supplementary Table 7. H3N2 binding to 6 µg mucins. Data used for Figure 2B. Values in the Figure are shown after subtracting signal from mucins without added virus.**

| **HNS LAM** | 0.01 | -0.03 | 0.02 | -0.01 | 0.03 | -0.02 | -0.06 | 0 | 0.04 | 0.02 |  |  |
| --- | --- | --- | --- | --- | --- | --- | --- | --- | --- | --- | --- | --- |
| **HNS LAM+H3N2** | 0.27 | 0.25 | 0.3 | 0.31 | 0.02 | 0.11 | 0.06 | 0.1 | 0.05 | 0.06 | 0.14 | 0.19 |
| **HNS LAM+H3N2+OC** | 0.26 | 0.28 | 0.6 | 0.37 | 0.16 | 0.21 |  |  |  |  |  |  |
| **LTS LAM** | 0.06 | -0.02 | -0.03 | -0.01 | 0.07 | -0.06 | -0.03 | 0.05 | -0.03 |  |  |  |
| **LTS LAM+H3N2** | 0.14 | 0.09 | 0.13 | 0.3 | 0.02 | 0.07 | 0.22 | 0.17 | 0.06 | 0.1 | 0.21 | 0.19 |
| **LTS LAM+H3N2+OC** | 0.31 | 0.42 | 0.57 | 0.3 | 0.21 | 0.16 |  |  |  |  |  |  |
| **LTS COPD LAM** | 0.06 | -0.08 | 0.02 | 0.05 | -0.04 | -0.03 | 0.03 | 0 | 0 | 0 |  |  |
| **LTS COPD LAM+H3N2** | 0.26 | 0.18 | 0.32 | 0.28 | 0.29 | 0.02 | 0.14 | 0.07 | 0.07 |  |  |  |
| **LTS COPD LAM+H3N2+OC** | 0.16 | 0.31 | 0.38 | 0.18 | 0.09 |  |  |  |  |  |  |  |
| **Pneunomia LAM1** | -0.13 | 0.07 | 0.06 | 0 | 0.08 | 0.07 | -0.15 | 0 | 0.02 | -0.02 |  |  |
| **Pneunomia LAM1+H3N2** | 0.26 | 0.23 | 0.27 | 0.07 | 0.26 | 0.06 | 0.46 | 0.05 | -0.03 | 0.01 | 0.29 | 0.16 |
| **Pneunomia LAM1+H3N2+OC** | 0.32 | 0.36 | 0.19 | 0.18 |  |  |  |  |  |  |  |  |
| **Pneunomia LAM2** | -0.04 | 0.06 | -0.02 | 0 | -0.1 | 0.1 | -0.04 | -0.03 | 0.02 | 0.04 |  |  |
| **Pneunomia LAM2+H3N2** | 0.38 | 0.19 | 0.2 | 0.26 | 0.15 | 0.2 | 0.12 | 0.08 | 0.11 |  |  |  |
| **Pneunomia LAM2+H3N2+OC** | 0.19 | 0.19 | 0.59 | 0.25 | 0.32 | 0.14 |  |  |  |  |  |  |
| **Oral MUC5B** | 0.02 | -0.04 | 0.02 | 0.18 | -0.14 | -0.04 | 0.03 | -0.04 | 0.01 |  |  |  |
| **Oral MUC5B+H3N2** | 0.14 | 0.11 | 0.23 | 0.4 | 0.26 | 0.04 | 0.07 | 0 |  |  |  |  |
| **Oral MUC5B+H3N2+OC** | 0.41 | 0.26 | 0.9 | 0.05 | 0.04 | 0.12 |  |  |  |  |  |  |
| **α2,3-HSA** | -0.02 | 0 | 0.02 | -0.02 | -0.13 | 0.15 | -0.02 | -0.02 | 0.02 |  |  |  |
| **α2,3-HSA+H3N2** | 0.17 | 0.18 | 0.18 | 0.14 | 0 | -0.11 | 0.08 | 0.19 | 0 | 0.25 | 0.15 |  |
| **α2,3-HSA+H3N2+OC** | 0.28 | 0.12 | 0.29 | 0.23 | 0.09 | -0.01 |  |  |  |  |  |  |
| **α2,6-HSA** | 0.07 | -0.12 | 0.05 | -0.11 | -0.16 | 0 | 0.01 | -0.01 |  |  |  |  |
| **α2,6-HSA+H3N2** | 0.27 | 0.22 | 0.21 | 0.33 | 0.14 | 0.1 | 0.6 | 0.1 | 0.15 |  |  |  |
| **α2,6-HSA+H3N2+OC** | 0.34 | 1.47 | 1.56 | 0.07 | 0.52 |  |  |  |  |  |  |  |

|  | **H1N1** | **H1N1+OC** | **H3N2** | **H3N2+OC** |
| --- | --- | --- | --- | --- |
| **HNS LAM** | 0.05 | 0.355 | 0.155 | 0.3133 |
| **LTS LAM** | 0.1058 | 0.445 | 0.1417 | 0.3283 |
| **LTS COPD LAM** | 0.1325 | 0.3083 | 0.1811 | 0.224 |
| **Pneunomia LAM1** | 0.055 | 0.41 | 0.1742 | 0.2625 |
| **Pneunomia LAM2** | 0.07833 | 0.3467 | 0.1878 | 0.28 |
| **Oral MUC5B** | 0.3527 | 0.615 | 0.1563 | 0.2967 |

**Supplementary Table 8. Average binding of virus with and without OC. Data used for Figure 3.**

**Supplementary Table 9. Data used for Figure 4.**

|  | **Average H1N1 binding** | **Average H3N2 binding** | **NeuAcα2-3/structure (%)** | **NeuAcα2-6/structure (%)** |
| --- | --- | --- | --- | --- |
| **HNS LAM** | 0.05 | 0.155 | 7.7275864 | 24.5161568 |
| **LTS LAM** | 0.1058 | 0.141667 | 18.96066 | 45.94765 |
| **LTS COPD LAM** | 0.1325 | 0.181111 | 41.18683 | 60.57997 |
| **Pneunomia LAM1** | 0.055 | 0.174167 | 76.911368 | 35.47140071 |
| **Pneunomia LAM2** | 0.07833 | 0.187778 | 79.126715 | 46.2768792 |
| **Oral MUC5B** | 0.3527 | 0.15625 | 22.60172 | 9.409905 |

| H1N1 | | | | | | H3N2 | | | | | |
| --- | --- | --- | --- | --- | --- | --- | --- | --- | --- | --- | --- |
| 10 µg mucin | 10 µg mucin (mock) | 3 µg mucin | 3 µg mucin (mock) | 1 µg Mucin | 1 µg mucin (mock) | 10 µg mucin | 10 µg mucin (mock) | 3 µg mucin | 3 µg mucin (mock) | 1 µg Mucin | 1 µg mucin (mock) |
| 53.55 | 134.43 | 81.53 | 107.18 | 133.69 | 97.67 | 89.62 | 126.39 | 74.86 | 95.68 | 79.88 | 112.46 |
| 63.50 | 83.13 | 64.49 | 94.67 | 95.47 | 119.67 | 66.04 | 172.99 | 79.75 | 103.35 | 78.30 | 94.59 |
| 77.48 | 136.39 | 98.01 | 87.23 | 101.03 | 117.50 | 63.32 | 67.58 | 161.54 | 179.80 | 75.05 | 89.02 |
| 62.23 | 98.72 | 88.57 | 84.64 | 82.91 | 109.67 | 77.83 | 101.70 | 107.44 | 120.03 | 70.69 | 85.93 |
| 63.41 | 149.92 | 61.12 | 109.81 | 88.63 | 112.31 | 64.03 | 75.30 | 127.37 | 96.38 | 64.42 | 117.22 |
| 59.28 | 65.01 | 93.82 | 84.60 | 96.75 | 91.74 | 62.98 | 82.59 | 103.76 | 67.32 | 48.72 | 61.59 |
| 59.72 | 69.13 | 78.71 | 162.63 | 103.25 | 98.11 | 72.52 | 91.35 | 214.02 | 69.16 | 55.82 | 114.45 |
| 62.51 | 66.52 | 59.65 | 151.20 | 93.91 | 84.61 | 69.63 | 108.52 | 106.95 | 109.35 | 62.29 | 75.79 |
| 54.91 | 116.82 | 61.44 | 69.56 | 77.19 | 70.14 | 63.67 | 90.20 | 94.12 | 101.41 | 95.72 | 123.08 |
| 50.95 | 79.92 | 70.11 | 48.48 | 78.80 | 98.57 | 57.97 | 83.38 | 88.35 | 57.52 | 61.49 | 125.86 |
|  |  |  |  | 103.51 | 91.45 |  |  |  |  | 130.24 | 103.39 |
|  |  |  |  | 182.11 | 173.01 |  |  |  |  | 163.98 | 58.65 |
|  |  |  |  | 122.57 | 153.94 |  |  |  |  | 112.70 | 94.45 |
|  |  |  |  | 122.51 | 72.62 |  |  |  |  | 96.02 | 129.35 |
|  |  |  |  | 76.27 | 100.17 |  |  |  |  | 99.37 | 136.82 |
|  |  |  |  | 143.08 | 73.58 |  |  |  |  | 78.14 | 116.34 |
|  |  |  |  | 90.57 | 75.09 |  |  |  |  | 144.48 | 53.74 |
|  |  |  |  | 149.95 | 114.68 |  |  |  |  | 95.36 | 97.97 |
|  |  |  |  | 75.59 | 77.19 |  |  |  |  | 65.03 | 100.34 |
|  |  |  |  | 58.45 | 68.27 |  |  |  |  | 66.17 | 108.95 |

**Supplementary Table 10. Normalized viral infection signal/DAPI (%). Data used for the Figure 5 graph.**

**Supplementary Table 10. Viral infection with OC. Normalized signal/DAPI (%). Data used for the Figure 6 graph.**

| H1N1 | | | | | | H3N2 | | | | | |
| --- | --- | --- | --- | --- | --- | --- | --- | --- | --- | --- | --- |
| 3 µg mucin | 3 µg mucin (mock) | 1 µg Mucin | 1 µg Mucin  (mock) | 0.3 µg Mucin | 0.3 µg Mucin  (mock) | 3 µg mucin | 3 µg mucin  (mock) | 1 µg Mucin | 1 µg Mucin  (mock) | 0.3 µg Mucin | 0.3 µg Mucin  (mock) |
| 104.87 | 99.63 | 114.43 | 161.40 | 149.68 | 64.42 | 85.01 | 63.37 | 78.39 | 66.81 | 69.37 | 75.61 |
| 75.69 | 132.69 | 76.83 | 102.55 | 127.99 | 106.13 | 97.90 | 158.92 | 58.68 | 72.54 | 120.02 | 58.58 |
| 71.11 | 134.78 | 77.83 | 85.85 | 63.59 | 126.11 | 96.14 | 89.76 | 54.60 | 113.77 | 110.87 | 35.84 |
| 58.64 | 92.48 | 50.44 | 108.76 | 103.50 | 98.47 | 82.82 | 80.51 | 106.44 | 103.03 | 96.00 | 78.23 |
| 128.24 | 107.91 | 71.39 | 110.70 | 84.72 | 130.49 | 120.47 | 131.60 | 145.09 | 149.50 | 107.93 | 215.71 |
| 69.42 | 82.27 | 135.08 | 108.47 | 168.39 | 98.76 | 85.09 | 50.25 | 92.65 | 59.25 | 92.31 | 108.47 |
| 61.73 | 68.56 | 74.13 | 93.95 | 165.10 | 69.42 | 117.31 | 102.74 | 100.85 | 80.58 | 82.99 | 89.72 |
| 71.95 | 76.35 | 67.65 | 61.63 | 71.42 | 52.55 | 103.86 | 99.85 | 109.09 | 71.80 | 115.43 | 93.81 |
| 40.69 | 57.74 | 82.28 | 78.62 | 112.09 | 113.07 | 109.50 | 104.49 | 58.56 | 148.04 | 96.47 | 124.22 |
| 50.06 | 147.59 | 64.96 | 88.07 | 64.52 | 140.59 | 106.04 | 118.51 | 90.09 | 134.68 | 130.66 | 119.82 |

**References**

1. Padra, M., et al., *Mucin Binding to Moraxella catarrhalis During Airway Inflammation is Dependent on Sialic Acid.* Am J Respir Cell Mol Biol, 2021.

2. Padra, M., et al., *Increased MUC1 plus a larger quantity and complex size for MUC5AC in the peripheral airway lumen of long-term tobacco smokers.* Clin Sci (Lond), 2020. **134**(10): p. 1107-1125.
